# Unmasking the ‘Asymptomatic’ COVID-19: A Nose Question

**DOI:** 10.3390/life12081248

**Published:** 2022-08-16

**Authors:** Andrea Mazzatenta, Anna Berardi, Gabriele Alessandro Novarria, Giampiero Neri

**Affiliations:** 1 Dipartimento di Neuroscienze, Imaging and Clinical Science, ‘G. d’Annunzio’ Chieti-Pescara University, Via dei Vestini 31, 66100 Chieti, Italy; 2 ENT Department, Istituto Clinico Città Studi, Via Jommelli 17, 20131 Milano, Italy

**Keywords:** COVID-19, SARS-CoV-2, smell test, asymptomatic, OST test

## Abstract

The new severe acute respiratory syndrome coronavirus 2 (SARS-CoV-2) virus has high infectivity, often masked by asymptomatic carriers, which allows it to spread rapidly and become a pandemic. Attempts to slow the pandemic at this stage depend on the ability to unmask asymptomatic carriers. The rapid diagnosis of active coronavirus disease 2019 (COVID-19) infection is one of the cornerstones of pandemic control, as the nasal cavity is the main gateway for SARS-CoV-2 entry and altered sense of smell is a feature of the current virus. In the present study, we therefore tested the olfactory threshold coupled with heart–lung parameters in subjects undergoing traditional molecular testing, resulting in a significantly different score between asymptomatic subjects and healthy controls. In total, 82% of asymptomatic positives showed olfactory impairment; of these, 46% had severe hyposmia and 7% had anosmia, while in the control 9% had severe hyposmia and 0% had anosmia, respectively, which agrees with heart rate, breathing rate, and blood pressure parameter variations. The olfactory test coupled with physiological parameters may help to identify asymptomatic people. In conclusion, our results suggest that most asymptomatic individuals could be unmasked by mass olfactory rapid threshold screening and then referred to traditional slower diagnostic tests.

## 1. Introduction

Since the explosive outbreak of COVID-19, the severity of the disease has been divided into four types: mild, moderate, severe, and critical cases [1]. However, most infections are so called ‘asymptomatic’ and can transmit the virus to others [2]. Asymptomatic infections have the same infectivity as symptomatic infections [3,4]. Asymptomatic infections refer to patients without any apparent clinical symptoms or distinctive signs, but present with the positive detection of nucleic acid of SARS-CoV-2 in samples derived from the reverse the transcriptase–polymerase chain reaction (RT-PCR) [5]. However, asymptomatic subjects can be categorized as: (*i)* incubatory carriers, who are able to transmit pathogens immediately following infection but prior to developing symptoms; (*ii)* convalescent carriers, who are capable of spreading disease following a period of illness, typically thinking themselves cured of the disease; and (*iii)* healthy carriers, who never exhibit signs or symptoms of the disease, yet are capable of infecting others, and are often considered to be the ‘classic’ asymptomatic carriers [6]. Most asymptomatic infected people do not seek medical assistance due to no obvious clinical signs and poor prevention awareness, contributing to the rapid spread of COVID-19 [7]. Despite researchers have made progress towards understanding the pathology, hitherto the mechanism of disease carrying is still unknown, how pathogen can remain dormant in a human for a period [8]. A better understanding of asymptomatic disease carriers, early recognition of an infected person and cutting off the route of transmission are key points to control COVID-19 [9]. Therefore, it is a great challenge to prevent and control this specific type of patient globally, which requires more attention worldwide [10].

In the plethora of early signs of COVID-19, viral spread causes a multi-system disease, including impairments in the brain, olfactory, and/or gustatory, although all olfactory and/or gustatory dysfunctions are not caused solely by the ongoing virus [11,12,13,14]. The olfactory system may be the most suitable system for detecting infection in the early stages, before the onset of symptoms or even in asymptomatic people [15]. Indeed, multiple cell types, present in the olfactory epithelium, express two host receptors, the proteases angiotensin-converting enzyme 2 (ACE2), transmembrane protease serine 2 (TMPRSS2), and neuropilin-1 receptor (NRP1), which facilitate the binding, replication, accumulation, and probably subsequent brain infection of SARS-CoV-2 through transneuronal/transynaptic pathways, as established for other SARS-CoV viruses [16,17,18,19].

It has been hypothesised that neuroinflammation is triggered by viral penetration from the nasal cavity or bloodstream; the result is activation of microglia, microthrombosis, coagulation, etc. (for review see [20,21,22]). Through histological assessment, persistent inflammation within the olfactory neuroepithelium, along with viral persistence, was shown [23]. Likewise, biopsies demonstrated the persistence of SARS-CoV-2 in the cells of the taste buds of the tongue [24].

The early detection of smell impairment in asymptomatic individuals is of diagnostic value because it is a possible early sign of infection [25]. Diagnostic systems for olfactory assessment are numerous; out of all of them, the most accurate test is the potential event-related olfactory test, which has already been applied for the diagnosis of dysosmia produced by COVID-19 [26]. However, this test requires considerable time, effort, equipment, and technical expertise. On the contrary, rapid tests allow the olfactory system of numerous subjects to be analyzed in a short time, at low cost and without specific equipment or specialized personnel; this can potentially prevent the further spread of the disease [25].

Applying rapid olfactory tests could be useful to understand the mechanism of chemoreceptive dysfunction in COVID-19 sufferers.

Accordingly, here we investigate the possibility of unmasking asymptomatic COVID-19 using a rapid, single-use, low-cost olfactory test, developed specifically for COVID-19 requirements [27], coupled with the physiological parameters of breath rate, heart rate, and blood pressure.

## 2. Materials and Methods

The retrospective study follows the Declaration of Helsinki and the Standards and Operational Guidance for Ethics of Health-Related Research with Human Participants [1,28], approved by the local ethic board and ‘G. d’Annunzio’ University and Local Sanitary Agency 2, with the code number colf01.2020 data, as of 9 November 2020.

The olfactory smart threshold test (OST, Asteria Healthcare) and the homemade two-element suprathreshold taste test (0.5 g/mL of sucrose and 0.5 g/mL of sodium chloride) were administered at the same time on sixty-seven subjects with no clinical symptoms of disease, who then voluntarily underwent the real-time polymerase chain reaction (RT-PCR) test of SARS-CoV-2 RNA at the Istituto Clinico Città Studi. The molecular test and the threshold test were administered simultaneously. Once the molecular result was obtained, the threshold result was examined.

The exclusion criterion was the presence or history of any diagnosed form of olfactory, nervous, cardiac, respiratory, renal, and hepatic disease.

**Figure 1:**
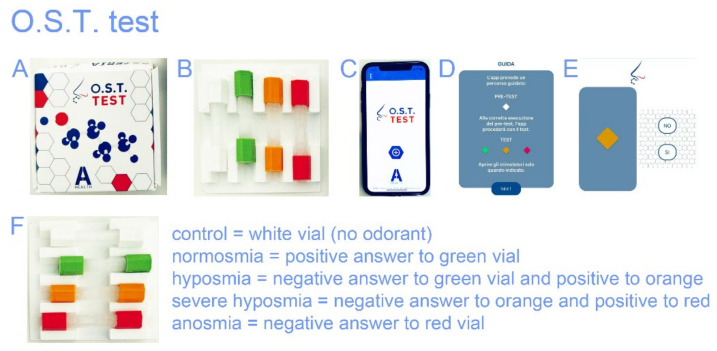
(**A**) OST test; (**B**) disposable vials; (**C**) the app used to perform the test; (**D**) the test guide; (**E**) the example of choice; (**F**) vials legend for the olfactory threshold assessment.

The OST test is based on the Connecticut Chemosensory Clinical Research Center (C.C.C.R.C.) threshold test [29] and the Italian population age phenotype threshold test [30]. The OST test uses a logarithmic scale of liquid n-butanol to assess positive answers: a green vial denotes normosmia, orange denotes hyposmia, and red denotes the severe hyposmia threshold, while no answer signals the presence of anosmia (Figure 1). The score was assigned to the color scale which ranged from #1 to 4, for normosmia, hyposmia, severe hyposmia, and anosmia, respectively. The white odorless vial is the test’s negative control. It is standardized, administered, and scored in a consistent manner. It was also validated for test–retest reliability [26,27]. The whole procedure took less than 2 min.

Physiological parameters, i.e., heart rate, breath rate, and blood pressure, were also collected using standard equipment.

One-way ANOVA statistical processing was used, with the α level set at 0.05, and *p* < 0.05 was considered significant. Commercial statistical software was used for all data and statistical analyses (IBM SPSS, Armonk, NY, USA; OriginLab Co., Northampton, MA, USA).

Age and gender were analyzed using MANOVA and post-hoc one-way ANOVAs, while the age was examined with olfactory phenotypes [30].

## 3. Results

The olfactory threshold test was assessed in sixty-seven subjects who showed no clinical symptoms and voluntarily underwent SARS-CoV-2 RNA real-time polymerase chain reaction (RT-PCR) testing. Among them were COVID-19-positive ‘asymptomatic’ subjects (mean age 53.8 ± 16.3 SD, range 23–87 years, 52% male and 48% female) and COVID-19-negative control subjects (mean age 51.5 ± 16.9 SD, range 25–75 years, 47% male and 53% female).

The sample was analyzed for gender and olfactory phenotype age-related MANOVA (*p* = 0.43, F_(3,63)_= 0.94). Post-hoc one-way ANOVAs include: olfactory phenotype vs. COVID-19 positivity, *p* = 0.63; gender vs. COVID-19 positivity, *p* = 0.12; olfactory phenotypes and gender vs. COVID-19 positivity, *p* = 0.86. The preliminary power analysis returns a value of 0.9 and a size effect ≥95%.

### 3.1. OST Test Score

**Figure 2:**
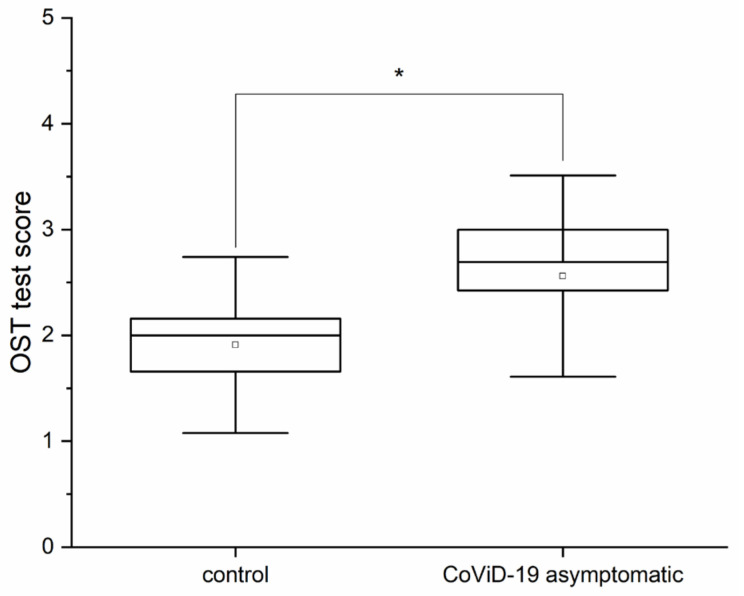
OST test score for olfactory threshold comparison between the control and asymptomatic COVID-19-positive subjects (* means significant for statistical analysis ANOVA *p* < 0.05).

An evaluation of the olfactory threshold, with the OST test, was carried out on subjects without clinical symptoms who voluntarily underwent molecular testing. The statistical analysis for the olfactory threshold, i.e., one-way ANOVA, returned significant differences (*p* < 0.05, F_(1,66)_ = 4.4) between the subjects who tested negative to the molecular test for SARS-CoV-2, i.e., the control subjects (mean OST test 1.9 ± 0.69 SD), and those who tested positive, i.e., the asymptomatic subjects (mean OST test 2.56 ± 0.9 SD) (Figure 2).

Furthermore, no significant difference was observed for the olfactory threshold between sexes (ANOVA *p* = 0.36, F_(1,66)_ = 0.87).

### 3.2. Heart Rate and Breath Frequency

**Figure 3:**
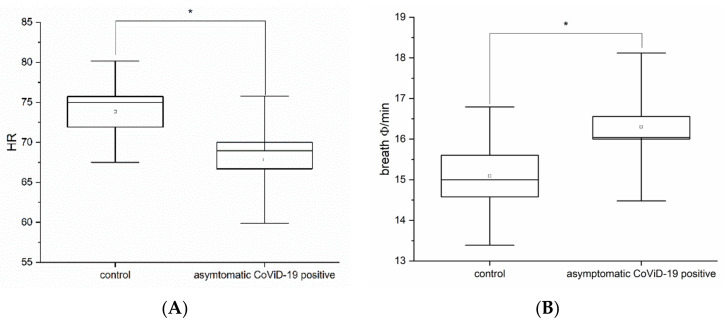
(**A**) Heart rate comparison in control vs. asymptomatic COVID-19-positive subjects (ANOVA *p* < 0.05). (**B**) Breath frequency comparison in control vs. asymptomatic COVID-19-positive subjects (* means significant for statistical analysis ANOVA *p* < 0.05).

By assessing the physiological parameters, significant differences were found in heart rate (HR) (*p* < 0.05, one-way ANOVA F_(1,66)_ = 5.5) between the control (mean HR 73.8 ± 6.3 SD) and the asymptomatic (mean HR 67.8 ± 7.9 SD) subjects (Figure 3A). Further, breath frequency (BF) between control (mean 15.1 ± 1.7 SD) and asymptomatic (mean 16.3 ± 1.8 SD) subjects was significantly different (*p* < 0.05, one-way ANOVA F_(1,66)_ = 4.1) (Figure 3B), while no significant differences for blood pressure (BP) (max *p* = 0.84, F_(1,66)_ = 0.04 and min *p* = 0.43, F_(1,66)_ = 0.62) were found.

### 3.3. Olfactory Threshold vs. Physiological Parameters

**Figure 4:**
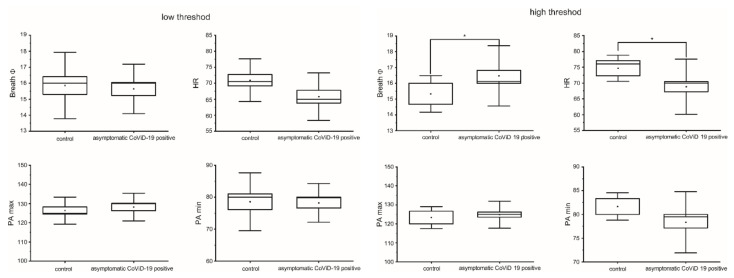
The (**left**) panel shows the box and whiskers of the physiological parameters grouped for the low olfactory threshold test score, indicating subjects with no or mild olfactory dysfunction. The (**right**) panel shows the same physiological parameters for the high-threshold score of subjects with severe olfactory disturbances or anosmia (* means significant for statistical analysis).

To explore a possible link between the olfactory threshold, other physiological parameters, and asymptomatic COVID-19-positive subjects, the olfactory threshold test score was used to segregate the physiological data into two groups: low threshold, i.e., subjects with mild or no olfactory dysfunction, and high threshold, i.e., subjects with severe olfactory impairment or anosmia (Figure 4). No difference was found in other physiological parameters of the low-threshold subjects; on the contrary, for the high-threshold subjects, a significant difference (*p* < 0.05) was found in BF and HR (F_(1,36)_ = 4.6 and 4.98, respectively).

### 3.4. COVID-19 High and Low Olfactory Threshold vs. BF and HR

**Figure 5:**
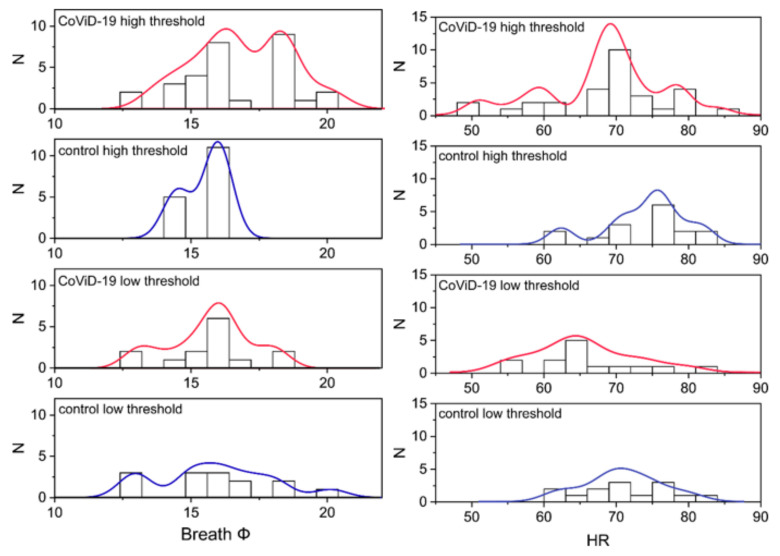
In the (**left**) panel, the distribution of BF is plotted for controls and asymptomatic COVID-19-positive high and low olfactory thresholds at the OST test score. In the (**right**) panel, the same is for HR.

Further analysis was performed on the frequency distribution to highlight differences in BF and HR, which correlated with the olfactory threshold test score (Figure 5). In the high-threshold group, the distribution of both physiological parameters diverged between the asymptomatic COVID-19 and the control subjects. The distribution fit showed a high threshold in asymptomatic (R^2^ = 0.69 for BF and R^2^ = 0.71 for HR), while there was a low threshold for the control (R^2^ = 0.79 for BF and R^2^ = 0.74 for HR) (R^2^ = 0.72 for BF and R^2^ = 0.68 for HR) (R^2^ = 0.52 for BF and R^2^ = 0.58 for HR). A comparison of high and low thresholds within the asymptomatic COVID-19-positive subjects showed a dramatic change in frequency distribution. These results suggest a potential subtle link between olfactory changes and physiological parameters, at least in terms of BF and HR.

## 4. Discussion

Olfactory impairment without other symptoms has been described as an isolated sudden-onset warning sign in confirmed COVID-19 [31]. Accordingly, we assessed the olfactory threshold in subjects without clinical symptoms who voluntarily underwent molecular testing for SARS-CoV-2.

Quantitative olfactory tests are essential to determine olfactory impairment and can also accurately monitor changes in function over time [32]. The comprehensive assessment of olfactory function includes the evaluation of various olfactory parameters, such as threshold, discrimination, identification, memory, electrophysiological, and metabolic activity, via specific tests [32].

Olfactory threshold tests are particularly useful for early olfactory screening. The old concept of testing with bottles, sticks, pens, etc., is unhygienic; moreover, given the volatility of odorants, re-use cannot guarantee stimulatory consistency. Advanced and rapid tests must be single-use and must involve standardized odor administration to ensure safety, reliability, and repeatability. There are essentially two commercial rapid smell tests [33,34] with substantial weaknesses, at least as far as applications in COVID-19 are concerned: they are reusable (so application in potentially infected patients is risky) and the stimulus concentration is inconstant over time with each use. The test used here, on the other hand, is disposable and ensures a constant stimulus concentration [27]. This test can also be self-administered because it does not require specific training, which is very useful to avoid contact with potentially infected patients [27,35].

Specifically, the stimuli used in identification tests are qualitative and identification suffers from cultural biases, cognitive ability, and olfactory ‘alphabetization’; for instance, a common stimulus of this tests whether the natural peppermint is a mixture of at least 20 pure odorants [36] and whether apple is a mixture of at least 37 odorants, which include n-butanol [37]. To explain the complexity of the qualitative tests, carvone, which is one of the odorants in peppermint, has two stereoisomers: one smells of caraway (the S-(+)) and the other of mint (the R-(−)). Again, isoamylacetate at varying concentrations gives a different perception that can vary from banana to pear, but is also contained in the smell of apple [37]. To try to overcome these obstacles, the rapid identification test was shaped by removing the odorants apple, turpentine, and garlic from the original test because they were identified by less than 55% of the normosmic validation cohort. Again, aniseed was eliminated because it was confused with liquorice [34,38].

The test employed here overcomes qualitative and quantitative limitations of identification test because they use the same stimulus, i.e., n-butanol, a pure odorant, on a logarithmic exponential growth scale, resulting in a purely quantitative stimulus [27]. The test is not limited by cultural biases, cognitive capability, and olfactory ‘alphabetization’, and is strengthened by statistical evaluation using test–retest analysis [27] and high-power analysis scores.

The other rapid threshold test comprised 20 smell “wands”, filled with half-log dilution steps of phenyl ethyl alcohol (PEA) in light USP-grade mineral oil, ranging from 10^–2^ to 10^–9^ vol/vol, which are employed to offer a comparison against five non-odor diluent blanks [33]. The delivery mechanism requires specific preparation, as well as the suggested method is a single-scale forced-choice paradigm that is difficult to apply in the clinical emergency frontier [33].

The OST test, on the other hand, overcomes these technical and methodical limitations and is easy to use and reproducible by anyone. Consequently, the threshold test used is the most suitable for the rapid clinical screening of patients potentially affected by COVID-19 rather than a discriminatory or identifying test.

However, it is important to point out that rapid tests for the assessment of olfactory function do not replace rapid molecular tests, but are an additional tool which provide greater possibilities in the search for asymptomatic subjects in combination with molecular tests. Although all testing methods have limitations of use, the rapid antigen test at the mass diagnostic level, mainly in the current scenario of several new SARS-CoV-2 variants, could generate false negatives [39]. The diagnostic accuracy of rapid antigenic tests is limited due to their sensitivity to variants (50%) [40].

In the third iteration of the Cochrane living review, summarizing the accuracy of point-of-care antigenic tests to detect current SARS-CoV-2 infection, several limitations are marked [41]. These include the unavailability of evidence for all commercial tests; poor compliance with manufacturers’ instructions; the fact that few tests meet the minimum requirements for acceptable sensitivity; the fact that tests have a steady decline in sensitivity as viral load decreases; the fact that tests have lower sensitivity in children; the fact that test sensitivity is lower in asymptomatic participants; and limited evidence for retesting strategies (test–retest analysis) [41]. When the prevalence is high, the impact of false negatives is dramatically important. The accuracy of rapid antigen tests in at-risk groups, e.g., hospital workers, is limited and may produce false negatives with a greater potential to create or increase the severity of existing outbreaks [41]. Rapid antigen tests show higher sensitivity in symptomatic patients than in asymptomatic ones, suggesting that viral load is a crucial parameter for antigen-based tests performed at points of care [42]. Accordingly, the sensitivity of the rapid antigenic tests was lower in asymptomatics, as more than half were falsely negative (58.7%), compared to symptomatics (84.2%) [43].

Therefore, the threshold test is particularly suitable for the rapid clinical screening of asymptomatic individuals potentially infected with COVID-19. It must be clear that this test is not intended to replace rapid antigenic tests, but could be useful in unmasking asymptomatic individuals. In a nutshell, this test stands to rapid antigenic testing as this one stands to RT-PCR laboratory tests.

SARS-CoV-2 acutely attacking the human olfactory system has been clearly shown in a cornerstone paper [44]. Sustentacular cells is the main target cell type in the olfactory epithelium, and leptomeningeal layers surrounding the olfactory bulb contain free viral RNA. Non-neuronal cells are sustentacular, regenerated throughout life from stem cells in the olfactory epithelium, and have glia-like properties, expressing ACE2 and TMPRSS2. Olfactory epithelial cells also express neuropilin-1, a potential cofactor facilitating SARS-CoV-2 cell entry and infectivity. There are intimate anatomical associations between sustentacular cells and olfactory sensory neurons, which could explain structural and/or physiological damage in olfactory sensory neurons when sustentacular cells are infected [44].

The viral RNA presence in leptomeningeal layers through the hematogenous route or the olfactory tract from the olfactory bulb to the cerebral cortex may contribute to olfactory dysfunction by perturbing signal propagation [44].

The authors leave the possibility open that OSNs may become infected and support viral replication in a subset of patients, or in certain disease courses or phases [44].

Consequently, this evidence seems to point to an etiology, at least in the initial stages, linked to peripheral damage in the olfactory epithelium. This may justify the rationale of using a threshold test, instead of a discriminative or identification test for screening purposes. Thus, this paper is innovative as, to the best of our knowledge, it is the only one to use a threshold test for screening. Furthermore, the threshold test provides a reliable, valid, inexpensive, and rapid clinical means of quantitatively assessing human olfactory sensitivity, which includes both peripheral and central impairments.

Consistent with other studies, we have also found that a decreased sense of smell is the initial symptom of the disease in asymptomatic subjects [45]. A likely bias would emerge from the fact that women present olfactory dysfunction more frequently than men [46,47,48,49]. One explanation could be the activation of X-chromosome-related toll-like receptors that could generate different inflammatory conditions and clinical courses following infection between men and women [50]. In contrast to these, and in agreement with other work [14,27], the results of our olfactory tests also showed that the effects of the virus on olfactory function is similar between sexes. Consequently, gender does not appear to be a bias for discriminating against asymptomatic individuals. Thus, fast olfactory threshold screening could be relevant for the control of COVID-19, as it could unmask asymptomatic subjects, independent of gender, as revealed by our experiment.

In addition, based on several studies showing the early alteration of other physiological parameters, we also studied heart rate, breathing rate, and blood pressure [51,52,53]. The disease is rather heterogeneous in its physiological manifestation, as observed in these studies, and it is currently not possible to distinguish SARS-CoV-2 infections from those caused by other viruses, as variations in physiological parameters are common to many respiratory infections [54]. Conversely, with previous studies, we found a slight significant decrease in heart rate, bradycardia, which agrees with a recent paper [53]. We could explain this result either by a number of subjects studied, which is, however, common to all studies, or by the fact that we refer to asymptomatic subjects and not to the early stages of the disease in our study, as in [51,52,53]. In addition, a slight significant increase in respiratory rate was also found, which agrees with previous studies conducted in the early phase of the virus [53,54], while no significant difference was found for blood pressure.

A potential correlation between altered olfactory thresholds, other physiological parameters, and asymptomatic COVID-19-positive subjects was positively investigated. Data from the physiological parameters, including heart rate and breath frequency, were segregated into two groups: the low olfactory threshold, i.e., subjects with mild or no olfactory dysfunction, and the high threshold, i.e., subjects with severe olfactory impairment or anosmia. Significantly, in the cluster with a high olfactory threshold, a correlation was found in both heart rate and breath frequency.

Subsequent analysis shows a dramatic change in frequency distribution between high and low olfactory thresholds between asymptomatic COVID-19-positive subjects and controls. These results suggest a potential subtle link between olfactory alterations and physiological parameters, particularly breath frequency and heart rate at the very least. These results agree with recent works [55,56]. A correlation between olfactory tests, as well alterations in physiological parameters (heart rate and breath frequency), suggests, the possibility of unveiling SARS-CoV-2 positivity, even in asymptomatic subjects.

The use of physiological parameters in connection with the olfactory threshold was used to strengthen the reliability of the olfactory threshold test, which can stand on its own and can be used during daily clinical practices.

This study is limited due to: (*i)* the low number of patients; (*ii)* the lack of oto-laryngological examinations and diagnoses of a hidden disease; and (*iii)* the lack of TDI use. Further studies need to exclude potential biases, such as the possible presence of other hidden diseases in the cohort of the studied sample or a larger sample (grouped by age or other factors, for example).

## 5. Conclusions

In conclusion, in the pandemic era of COVID-19, olfactory changes, even without other upper respiratory tract infections or other symptoms, could be early signs of SARS-CoV-2. These findings reinforce the need for a rapid, reliable, inexpensive, and manageable olfactory test for anyone, as a first-level diagnostic flowchart, in order to contain the spread of COVID-19 and its new pulsatile recurrent variants. Thus, in asymptomatic COVID-19-positive subjects, the diagnostic value of detecting olfactory impairment coupled with other physiological parameters is a possible concealed sign which can unmask the virus.

## Data Availability

All data information can be found in the paper and is preserved in the repository of ENT Department, Istituto Clinico Città Studi.
